# Site occupancy by American martens and fishers in temperate deciduous forests of Québec

**DOI:** 10.1093/jmammal/gyac092

**Published:** 2022-12-09

**Authors:** Pauline Suffice, Marc J Mazerolle, Louis Imbeau, Marianne Cheveau, Hugo Asselin, Pierre Drapeau

**Affiliations:** Institut de recherche sur les forêts, Université du Québec en Abitibi-Témiscamingue, Rouyn-Noranda, Québec, Canada; Département des sciences du bois et de la forêt, Université Laval, Québec City, Québec, Canada; Institut de recherche sur les forêts, Université du Québec en Abitibi-Témiscamingue, Rouyn-Noranda, Québec, Canada; Ministère des Forêts, de la Faune et des Parcs du Québec, Québec City, Québec, Canada; École d’études autochtones, Université du Québec en Abitibi-Témiscamingue, Rouyn-Noranda, Québec, Canada; Département des sciences biologiques, Université du Québec à Montréal, Montréal, Québec, Canada

**Keywords:** American marten, camera traps, fisher, interspecific interactions, local knowledge, site occupancy, temperate deciduous forest, trappers, appareils photo à déclenchement automatique, forêts tempérées feuillues, interactions interspécifiques, martre d’Amérique, occupation de sites, pékan, savoirs locaux, trappeurs

## Abstract

Interspecific interactions can mediate site occupancy of sympatric species and can be a key factor in habitat use patterns. American martens (*Martes americana*) and Fishers (*Pekania pennanti)* are two sympatric mesocarnivores in eastern North American forests. Due to their larger size, fishers have a competitive advantage over martens. We investigated site occupancy of martens and fishers in temperate deciduous forests of Québec, an environment modified by forest management and climate change. We formulated hypotheses on the spatial distribution of the studied species based on the knowledge of local trappers and on the scientific literature regarding forest cover composition, habitat fragmentation, and competitive relationships. We used a network of 49 camera traps monitored over two fall seasons to document site occupancy by both species. We used two-species site occupancy models to assess habitat use and the influence of fishers on martens at spatial grains of different sizes. None of the habitat variables that we considered explained site occupancy by fishers. Availability of dense old coniferous stands explained the spatial distribution of martens both at the home range grain size and at the landscape grain size. We identified the characteristics of habitat hotspots based on the knowledge of trappers, which highlighted the importance of stand composition, height, age, and canopy closure. The characteristics of habitat hotspots for martens in temperate deciduous forests refine the habitat suitability model for American martens that was originally developed for boreal forests of Québec.

## Abstract (fr)

Les interactions interspécifiques peuvent affecter l’occupation de sites par des espèces sympatriques et jouer un rôle clé dans leur utilisation des habitats. La martre d’Amerique (*Martes americana*) et le pékan (*Pekania pennanti*) sont deux mésocarnivores sympatriques des forêts de l’est de l’Amérique du Nord. En raison de sa grande taille, le pékan est un compétiteur dominant de la martre. Nous avons étudié l’occupation des sites par la martre et le pékan dans la forêt tempérée feuillue du Québec, un environnement modifié par l’aménagement forestier et les changements climatiques. Nous avons formulé des hypothèses sur la répartition spatiale des espèces étudiées en nous basant sur les connaissances des trappeurs locaux et sur la littérature scientifique en ce qui a trait à la composition du couvert forestier, à la fragmentation de l’habitat, et aux relations de compétition. Nous avons utilisé un réseau de 49 appareils photo à déclenchement automatique pendant deux automnes pour documenter l’occupation des sites par les deux espèces. Nous avons utilisé des modèles d’occupation de sites à deux espèces afin d’évaluer l’effet de la présence du pékan sur l’utilisation de l’habitat par la martre à des échelles spatiales de résolutions variables. Aucune des variables d’habitat que nous avons prises en compte n’explique l’occupation des sites par les pékans. La disponibilité de vieux peuplements denses de conifères explique la répartition spatiale de la martre aux échelles spatiales du domaine vital et du paysage. Nous avons développé un indice d’habitat potentiel basé sur les connaissances des trappeurs, qui a mis en évidence l’importance de la composition, de la hauteur, de l’âge et de la densité des peuplements. Cet indice affine, pour les forêts tempérées feuillues du Québec, le modèle de qualité de l’habitat de la martre d’Amérique originellement élaboré pour la forêt boréale.

Human disturbances and climate change influence habitat use and the composition of communities (Graham and Grimm 1990; Lodge 1993; Jensen and Humphries 2019), which may affect long-term interspecific interactions as species respond, for example, by shifting their geographic distributions, in individualistic ways (Vanak and Gompper 2010; Fisher et al. 2013; Sweitzer and Furnas 2016). American martens (*Martes americana*, “martens,” hereafter) and fishers (*Pekania pennanti*) are solitary and elusive forest mesocarnivores. Their habitat use is similar in many respects. Fishers and martens use habitats with complex horizontal and vertical physical structures, in stands consisting of coniferous and deciduous tree species. Forest complexity including snags and coarse woody debris provide fishers and martens with access to prey, resting and denning sites, as well as protection from predators (Buskirk and Powell 1994; Powell et al. 2003; Gilbert et al. 2017).

Fishers and martens use habitat based on characteristics typical of late successional forests (large trees, complex vertical and horizontal structure; Raley et al. 2012; Gilbert et al. 2017). Yet, fishers apparently use a larger range of forest cover types in more diversified spatial configurations, including those affected by habitat fragmentation, than do martens (Sauder and Rachlow 2015). Given that fisher movements are constrained by deep and uncompacted snow, the species is especially associated with coniferous stands that intercept snow before it accumulates on the ground (Raine 1983; Krohn et al. 1995; Manlick et al. 2017). In contrast, martens are sensitive to loss of mature and old-growth habitats resulting from timber harvesting (Thompson 1991; Potvin et al. 2000; Cheveau et al. 2013).

Habitat use can also be shaped by interactions between species (Dunning et al. 1992; Wiens et al. 1993; Amarasekare 2003). Mesocarnivores are particularly affected by the presence of larger sympatric species (Donadio and Buskirk 2006; Wang et al. 2015; Zielinski et al. 2017). For example, marten distribution is influenced by interactions with predators (Krohn et al. 1995; Fisher et al. 2013; Suffice et al. 2017). Fishers are larger than martens and may competitively exclude martens from certain areas (Thomasma 1996; Fisher et al. 2013). Coexistence can occur at a landscape scale, that is, within a local community defined as a set of species that occupy a spatial extent of the landscape within which routine feeding and breeding activities and species interactions such as competition and predation occur (Amarasekare 2003). However, coexistence at a landscape scale also allow local exclusion if two competing species share the same ecological niche (Hardin 1960). Martens and fishers are sympatric across several portions of North America (Fisher et al. 2013; Manlick et al. 2017; Croose et al. 2019), such as in temperate deciduous forests of Québec (eastern Canada). Suffice et al. (2020) reported that sales of fisher pelts increased over the past 30 years in Québec, whereas sales of marten pelts decreased during the same period. This pattern may be the result of competition between the two species in eastern Canada, which might not be the case elsewhere in the sympatric zone (Gompper et al. 2016; Croose et al. 2019).

We used camera traps in temperate deciduous forests of Québec to assess habitat occupancy of fishers and martens and to establish whether evidence is consistent with the hypothesis that there is competitive exclusion by fishers on martens in our study area where both species are sympatric. We also combined the knowledge of local trappers and the scientific literature to elucidate the effects of habitat variables on fisher and marten co-occurrence, and identified the characteristics of habitat hotspots for martens and fishers (Suffice et al. 2017; Webb et al. 2019).

We hypothesized that, within our study area, the spatial distribution of the studied species would be explained by forest cover composition. We predicted that mixedwood and coniferous stands would increase occupancy for both species. In contrast, we predicted that occupancy by martens in deciduous stands would decrease due to the lack of sufficient protection from predators, while occupancy by fishers would not be affected. At the home range grain size, we hypothesized that the spatial distribution of fishers and martens would be affected by competitive exclusion relationships, with martens avoiding sites preferred by fishers. We furthermore hypothesized that the spatial distribution of fishers and martens would be affected by habitat fragmentation such that: (1) areas with high road densities should be avoided by both species (Gompper et al. 2016); and (2) forest edges should attract fishers (Powell 1979; Sauder and Rachlow 2015), where they could find a wide range of prey species (Jones 1991; Jones and Garton 1994), whereas martens would prefer closed-canopy forests to avoid predators.

## Materials and Methods

### Study area

We assessed site occupancy by martens and fishers in the Témiscamingue region of western Quebec, Canada. The study area is located mainly in the sugar maple‒yellow birch bioclimatic domain, but extends northward into the balsam fir‒yellow birch domain (Bélanger et al. 1992). The sugar maple‒yellow birch domain is the northernmost part of temperate deciduous forest of Québec, where yellow birch (*Betula alleghaniensis*) is one of the main species with sugar maple (*Acer saccharum*). American beech (*Fagus grandifolia*), red oak (*Quercus rubra*), eastern hemlock (*Tsuga canadensis*), and eastern white pine (*Pinus strobus*) are the other major species in these forests. Windthrow, which creates canopy gaps by knocking down one or a few trees, is the main disruptive agent structuring natural forest dynamics in the sugar maple‒yellow birch domain (Drever et al. 2006; Després et al. 2017). The balsam fir‒yellow birch domain represents the transition from temperate to boreal forests. These mixed stands are mainly composed of balsam fir (*Abies balsamea*) with yellow birch, white spruce (*Picea glauca*), and eastern white cedar (*Thuja occidentalis*). Outbreaks of the spruce budworm (*Choristoneura fumiferana*) and wildfires are the two main drivers of natural forest dynamics (Bouchard et al. 2008). Forest management, mostly with partial cuts, is also an important driver of habitat change in the study area (Danneyrolles et al. 2016). In addition, the region is affected by climate change, notably by a decrease of snowpack thickness and an increase of spring rainfall (Logan et al. 2011). The study area straddles the Ontario border to the west, which is characterized by forests that are similar in composition to those of Témiscamingue. Unlike other areas where martens and fishers are sympatric (Fisher et al. 2013; Gompper et al. 2016; Sweitzer and Furnas 2016), Témiscamingue lacks a strong elevational gradient (176–376 m).

Trapping is particularly important in the Témiscamingue region, where yields (number of pelts sold/100 km^2^) of marten and fisher pelts are among the highest in Québec (Suffice et al. 2020). The organization of trapping in the region is mainly through traplines, each of which is held by a single trapper, who is responsible for the sustainable harvest of the trapline for several years. The trapping season starts in mid-October and coincided with camera-trap monitoring at our sites.

### Species detection design

We selected 49 sites along roadways (paved and logging roads) that were accessible throughout the year. Based on the size of the home range of fisher females that were monitored in a region adjacent to our study area (29.9 ± 5.19 km²; Tully 2006), sites were systematically established every 5.8 km along the most accessible forest roads to ensure their independence. At each sampling site, we installed a motion-detection infrared camera (Bushnell Trophy Cam HD Aggressor No-Glow, 119776C). We used large-size portions of moose from a butcher shop (bones, cutting waste, and skin) to simulate a cervid carcass on the ground. The bait was deployed about 5 m in front of the camera at each site and was equivalent to half a moose (half of the rib cage and two legs) without the meat. Logs greater than 15 cm in diameter were placed on top of the bait to prevent it from being moved by wolves (*Canis lupus*), coyotes (*Canis latrans*), or black bears (*Ursus americanus*). The bait retained a strong odor over the course of the study. In addition to the bait, a long-distance olfactory lure was placed on a tree within the field of view of the camera (Forget’s XLDC lures, Sherbrooke, Quebec, Canada).

Camera traps were operated from 11 October to 6 December 2015 and from 19 October to 12 December 2016. Sites were visited every 1 or 2 weeks and rebaited if needed. All photographs were inspected to determine detections of each species during each day (between two sunrises). The information was compiled into detection histories for each site and each year.

### Habitat

Relationships between wildlife and habitat are scale-dependent (Levins 1968; Wiens 1989; Hall et al. 1997). We studied habitat use at grain sizes corresponding to marten and fisher home ranges. We used ArcGIS 10.5 (Environmental Systems Research Institute, Redlands, California) to georeference each sampling site to the recent digital forest cover maps from Quebec (Lemieux et al. 2015) and Ontario (Ontario Ministry of Natural Resources 2009). The maps included updates of natural (windthrow, insect outbreaks, and wildfire) and anthropogenic (partial or total cuts) disturbances. We measured habitat suitability in three ways: (1) using habitat composition by tree height class and species composition; (2) considering spatial fragmentation as revealed by road and edge densities (km/km^2^); and (3) using a habitat hotspot index based on the knowledge of local trappers.

We measured habitat composition and fragmentation variables around the cameras using the methodology developed by Potvin et al. (2000) as well as empirical local knowledge shared by trappers (Suffice et al. 2017). We grouped forest stands by combining two criteria considered important for forest mustelids (Potvin et al. 2000; Cheveau et al. 2013): height classes (7–12 m, >12 m) as a proxy of structure and composition based on dominance (deciduous: <25% coniferous; mixed: 25–75% coniferous; coniferous: >75% coniferous). In our study area, a very small proportion of the forest cover was in the 7- to 12-m height class (4–5%, Supplementary Data SD1). Thus, we analyzed the effect of three different habitats: stands that were higher than 12 m and mostly deciduous (Decid12); mixed (Mixed12); coniferous (Conif12).

We calculated a habitat index that combines parameters included in the habitat suitability model for marten developed by Québec government (FAPAQ 2000) and trapper knowledge. We compiled records of 41 trapper interviews that provided empirical knowledge on successful trapping habitats of martens in temperate forests (Suffice et al. 2017). We translated the description that came up most often during the interviews into stand categories available in the forest inventory maps of the Quebec government. During the interviews, trappers stated that martens preferentially used mixedwood or mature coniferous stands that were “dirty and dense” in our study region, corresponding to uneven-aged multistage forests (Suffice et al. 2017). Trappers identified deciduous stands as being used by martens to hunt and travel to other areas. In the same study region, trappers indicated that fishers also use mixedwood and coniferous stands, but these stands were not as dense nor as old as those used by martens (Suffice et al. 2017). We considered combinations of variables defining habitat Hot Spots for Fishers (HSF) and Hot Spots for Martens (HSM) based on the knowledge of trappers. HSF corresponded to coniferous or mixedwood stands taller than 7 m with a canopy closure ≥ 25%. We expected that site occupancy by fishers would increase with the proportion of HSF around a site. HSM consisted of mixedwood or coniferous stands >12 m in height and >90 years old, with a canopy closure ≥ 60%. We expected that site occupancy by martens would increase with the proportion of HSM around a site. We tested the capacity of HSF and HSM to increase our efficiency to explain occupancy patterns by the two species. Given the low number of sites occupied by the species and the limited number of sites sampled, we had to restrict the number of variables in any given model. We focused on stand height and composition, the variables most often used to guide forest management in Quebec (Potvin et al. 2000) as well as those included in the HSF and HSM combinations in order to benefit from the expertise shared by the trappers. Indeed, compared to the models based solely on stand height or composition, those derived from trapper knowledge also included stand density and age, highlighting the importance of data complementarity (Supplementary Data SD1). We measured forest fragmentation by calculating the density (km/km^2^) of edges between forest stands > 4 m in height and open environments (vegetation ≤ 4 m in height). We also calculated a road density index (km/km^2^). Road lengths were weighted according to their vocation (road class influencing the type of vehicle that can travel on it), width, and rolling surface. Forest roads (narrow and unpaved) had a weight of 1, access and collector roads had a weight of 2, and numbered roads (wide and paved) had a weight of 3.

We quantified the respective proportions of all forest characteristics around each sampling site at different spatial grain sizes using radii of 0.5, 1, 3, and 5 km, which represented areas of 0.78, 3.1, 28.3, and 78.5 km^2^, respectively. Fisher et al. (2011) tested a range of 20 spatial grain sizes and found that fisher site occupancy was best predicted by habitat quantified within a 0.5-km radius. Since no similar analysis was published to determine the best spatial grain size for predicting marten site occupancy, we used the 0.5-km grain size around the cameras to quantify site occupancy by both species, thus facilitating comparison. Radii of 1, 3, and 5 km, respectively, represented the home range sizes of martens (Dumyahn et al. 2007; Godbout and Ouellet 2008; Jensen 2012), female fishers, and male fishers (Powell et al. 2003; Tully 2006).

### Detection covariates

Because the probability of detecting the species by camera is imperfect, we considered variables potentially influencing detection. We used meteorological data interpolated from the three nearest Environment Canada weather stations to estimate daily weather measures specific to each site (BioSIM 11.4.6.0: Régnière and St-Amant 2007; Régnière et al. 2017). We included the quantity of rainfall (Rain) and daily minimum temperature (TempMin) as explanatory variables that could affect the probability of detecting the species. We expected that low temperature (below −20°C) would limit the movements of martens and fishers and, therefore, have a negative effect on their detection probability (Zielinski 2000; Weir et al. 2005). Furthermore, rain could reduce the effectiveness of the bait (carcass, skin, and lure) (Pawlina and Proulx 1999). We also considered day-of-year (JulianDay) and the number of days since deployment of the lure (LureDay) as additional variables potentially influencing the probability of detection. We expected that detection probability would be higher at the end of the season (December) when access to food is more limited and our bait more appealing (Moriarty et al. 2015). We further predicted that lure effectiveness would diminish with the number of days following its application.

### Statistical analyses

We constructed single-season models of co-occurrence of the two studied species (MacKenzie et al. 2004). The raw data consisted of the detection histories of each species at each site (i.e., 0s and 1s indicating nondetection or detection for each day of sampling, respectively). Detection histories spanned 26–57 days. Data for each year were superimposed and treated as being independent (Fuller et al. 2016; Linden et al. 2017) and a year effect was included in all models on occupancy and detection probability parameters. We quantified the effect of forest characteristics on the occupancy of each species, while considering the presence of its competitor, using the conditional parameterization approach of Richmond et al. (2010). This model formulation treats one of the two species as being dominant (A = fishers) and the other as subordinate (B = martens). Because fishers are dominant over martens (Krohn et al. 1995), we predicted that the probability of marten occurrence would decrease in the presence of fishers. We used the notation of Richmond et al. (2010) to define the model parameters. Briefly, we estimated the probability of site occupancy by fishers (ψ^A^), and the probability of site occupancy by martens in the absence (ψ^Ba^) or in the presence of fishers (ψ^BA^). In order to reduce the number of estimated parameters in the models, we applied constraints to the detection probabilities. Specifically, we considered the probability of detecting fishers to be independent of the presence of martens with the constraint *p*^A^ = *r*^A^, where *p*^A^ is the probability of detecting fishers when martens are absent, and *r*^A^ is the probability of detecting fishers when both species are present. We considered the probability of detecting martens to be independent of the presence and detection of fishers (*p*^B^ = *r*^Ba^ = *r*^BA^).

To test our hypotheses, we considered two scenarios of marten occupancy relative to fisher presence, that is, either dependence of marten occupancy on fisher presence (ψ^BA^ ≠ ψ^Ba^) or independence of marten occupancy on fisher presence (ψ^BA^ = ψ^Ba^). For each of these two marten occupancy scenarios, we constructed 25 candidate models (Table 1) that included variables not strongly correlated with one another (|*r*| < 0.7). Each of the two marten occupancy scenarios included a null model consisting exclusively of an effect of Year on all parameters (Supplementary Data SD1). We considered three habitat scenarios consisting of (a) habitat composition by height class and species composition; (b) spatial fragmentation including roads and edges; and (c) habitat indexes (HSM and HSF) based on local knowledge. These three habitat scenarios were tested at four spatial grain sizes based on radii of 0.5, 1, 3, and 5 km around each site, yielding a total of 12 habitat–grain size combinations. Each of these 12 habitat–grain size combinations was tested for each of the two marten occupancy scenarios (dependent or independent of presence of fisher). The occupancy models combined each of the 12 habitat–grain size combinations with one of two sets of detection probability variables consisting of (1) minimum temperature, rain, and number of days since the application of the lure; or (2) minimum temperature, rain, and Julian date. Thus, our candidate model set included a total of 50 models (2 martens occupancy ­scenarios × 12 habitat–grain size combinations × 2 detection scenarios = 48 models + 2 null models). Parameters were estimated by maximum likelihood using the PRESENCE 2.12.17 software (USGS, Patuxent Wildlife Research Center, Laurel, Maryland; MacKenzie et al. 2006). Candidate models were compared for all spatial grain sizes combined and for each spatial grain size separately using the Akaike Information Criterion (AIC_c_) for small samples (Burnham and Anderson 2002). We performed multimodel inference in R with the AICcmodavg package using the shrinkage estimator and unconditional 95% confidence intervals for each parameter of interest (Burnham and Anderson 2002; Mazerolle 2020; R Core Team 2021). We presented the predictors and their associated standard errors, which were calculated using the delta method (Oehlert 1992; MacKenzie et al. 2006).

**Table 1.** —Variables used in occupancy models to explain the presence and detection probabilities of marten and fisher in temperate forests of western Quebec. HSF = Hot Spots for Fishers; HSM = Hot Spots for Martens.

Variable	Description	Range	Units
Year	Year of monitoring	2015–2016	year
Decid12	Proportion of stands ≥ 12 m in height that were mainly deciduous	0–73.64	%
Mixed12	Proportion of stands ≥ 12 m in height that were mainly mixedwood	0–79.78	%
Conif12	Proportion of stands ≥ 12 m in height that were mainly coniferous	0–73.22	%
Roads	Density index of roads weighted according to their class	0.08–0.30	km/km^2^
Edges	Interface length between stands > 4 m in height and environments ≤ 4 m in height	1.98–9.34	km/km^2^
HSF	Habitat hotspots for fisher identified by trappers (mixedwood or coniferous stands ≥ 7 m in height with a canopy closure ≥ 25%)	0–40.01	%
HSM	Habitat hotspots for marten identified by trappers (mixedwood or coniferous stands ≥ 12 m high and ≥90 years old, with a canopy closure ≥ 60%)	0–98.00	%
TempMin	Daily minimum temperature	−23 to 11	°C
Rain	Quantity of rainfall	0–20	mm
JulianDay	Day-of-year	11 October 11 to 12 December	day
LureDay	Number of days since deployment of the lure	0–26	day

## Results

### Habitat

Forests > 12 m in height accounted for 68–71% of the area characterized over the four spatial grain sizes (Supplementary Data SD2). Proportions of our two habitat hotspots (HSF and HSM) varied little across spatial grain sizes, but variability among sites at a given spatial grain size was larger at small compared to large spatial grain sizes (Supplementary Data SD3). The average HSF proportion was five times that of HSM. Edge densities were comparable across spatial grain sizes, but variability decreased with increasing grain size (Supplementary Data SD4). The weighted road index was similar among the three smallest spatial grain sizes, but lower at the larger grain size (Supplementary Data SD5).

### Sampling conditions

Estimated daily rainfall averaged 3 mm (±*SD*: 4, [minimum, maximum: 0, 18]) in 2015 and 1 mm (±2, [0, 20]) in 2016. Cumulative rainfall for the camera-trapping period was 117 mm (±20, [63, 144]) in 2015 and 62 mm (±5, [36, 71]) in 2016. Daily minimum temperature averaged −1°C (±4, [−17, 11]) in 2015 and −2°C (±5, [−23, 8]) in 2016. The number of days between two lure applications averaged 9 days in 2015 (±7, [0, 26]) and 2016 (±6, [0, 25]).

### Detection

**Fig. 1.**
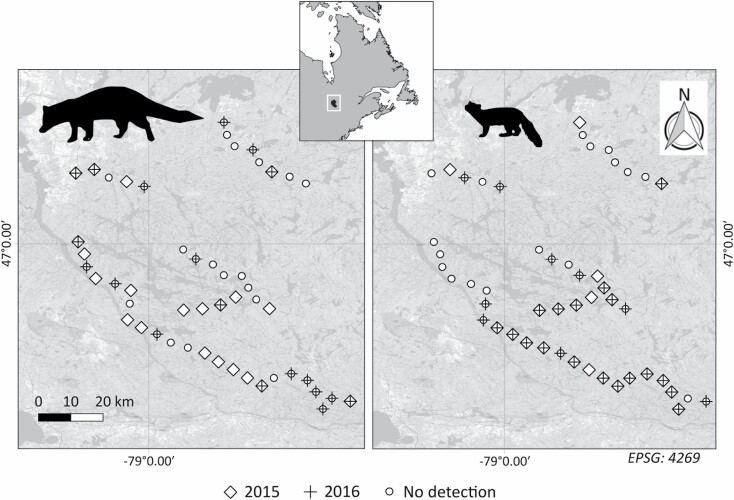
—Detection of fisher (left) and marten (right) by camera traps at 49 sites in Témiscamingue, Quebec, Canada (eastern North American temperate deciduous forest) in falls 2015 and 2016. Diamonds represent sites where at least one individual was detected in 2015. Crosses indicate sites where at least one individual was detected in 2016. Circles indicate sites without detection.

Effort (number of sampling days) at the 49 camera-trap stations was 2,216 (average days per camera trap ± *SD*, [minimum, maximum]: 45 ± 9 days, [26–57]) in 2015 and 2,566 (52 ± 3 days, [34, 53]) in 2016. Fishers were detected by camera traps at 21 sites (43%) in 2015 and 19 sites (39%) in 2016, for a total of 33 different sites (67%), including 7 (14%) sites with detection in both years (Fig. 1). Martens were detected at 23 sites (47%) in 2015 and 27 sites (55%) in 2016, for a total of 32 different sites (65%), 18 (37%) of which detected martens in both years. The two species were detected at 10 sites (20%) each year, including two sites where both species were detected in both years. In total, we recorded 100 fisher detections out of 2,216 camera-days in 2015 (4.5%), with a maximum of 19 days at the same site, and in 105 out of 2,566 camera-days in 2016 (4.1%), with a maximum of 20 days at the same site (Supplementary Data SD6a). Martens were detected on 185 days in 2015 (8.3%) with a maximum of 29 days at the same site, and on 318 days in 2016 (12.4%) with a maximum of 39 days at the same site (Supplementary Data SD6b).

### Covariate effects on site occupancy and species ­detection

For all spatial grain sizes, models considering the probability of marten occupancy independent of fisher presence (ψ^BA^ = ψ^Ba^) systematically had more support than models considering an effect of fisher presence. Indeed, models allowing marten occupancy to vary with the presence or absence of fishers had very low support (sum of Akaike weights = 0.01). As a result, the effect of habitat variables on site occupancy by martens did not depend on the presence of fishers. Models including HSF and HSM and forest cover of stands > 12 m in height (Decid12, Mixed12, and Conif12) always had greater support compared to models considering the spatial fragmentation of habitats (Table 2). HSM and HSF better explained site occupancy by martens than did spatial fragmentation (Table 3).

**Table 2.** —Ranking of two-species occupancy models for all spatial scales combined and for each spatial scale separately according to their Akaike weights (*w*_*i*_). Only models with Akaike weights ≥ 0.01 are presented. HSF = Hot Spots for Fishers; HSM = Hot Spots for Martens.

Scale	Model	*K*	AIC_c_	Δ_*i*_ AIC_c_	*w*_*i*_
All	ψ^A^ (Year + HSF_5 km_ + HSM_5 km_) ψ^BA^ = ψ^Ba^ (Y + HSF_5 km_ + HSM_5 km_) *p*^A^ (Year + TempMin + Rain + JulianDay) *p*^B^ (Y + TempMin + Rain + JulianDay)	18	4,014.51	0.00	0.46
	ψ^A^ (Year + HSF_3 km_ + HSM_3 km_) ψ^BA^ = ψ^Ba^ (Year + HSF_3 km_ + HSM_3 km_) *p*^A^ (Year + TempMin + Rain + JulianDay) *p*^B^ (Year + TempMin + Rain + JulianDay)	18	4,014.59	0.07	0.45
	ψ^A^ (Year + Decid12_5 km_ + Mixed12_5 km_ + Conif12_5 km_) ψ^BA^ = ψ^Ba^ (Year + Decid12_5 km_ + Mixed12_5 km_ + Conif12_5 km_) *p*^A^ (Year + TempMin + Rain + JulianDay) *p*^B^ (Year + TempMin + Rain + JulianDay)	20	4,018.35	3.84	0.07
	ψ^A^ (Year + HSF_5 km_ + HSM_5 km_) ψ^BA^ (Year + HSF_5 km_ + HSM_5 km_) ψ^Ba^ (Year + HSF_5 km_ + HSM_5 km_) *p*^A^ (Year + TempMin + Rain + JulianDay) *p*^B^ (Year + TempMin + Rain + JulianDay)	22	4,023.02	8.50	0.01
0.5 km	ψ^A^ (Year + Decid12_0.5 km_ + Mixed12_0.5 km_ + Conif12_0.5 km_) ψ^BA^ = ψ^Ba^ (Year + Decid12_0.5 km_ + Mixed12_0.5 km_ + Conif12_0.5 km_) *p*^A^ (Year + TempMin + Rain + JulianDay) *p*^B^ (Year + TempMin + Rain + JulianDay)	20	4,039.16	0.00	0.72
	ψ^A^ (Year + HSF_0.5 km_ + HSM_0.5 km_) ψ^BA^ = ψ^Ba^ (Year + HSF_0.5 km_ + HSM_0.5 km_) *p*^A^ (Year + TempMin + Rain + JulianDay) *p*^B^ (Year + TempMin + Rain + JulianDay)	18	4,041.32	2.17	0.24
	ψ^A^ (Year + Roads_0.5 km_ + Edges_0.5 km_) ψ^BA^ = ψ^Ba^ (Year + Roads_0.5 km_ + Edges_0.5 km_) *p*^A^ (Year + TempMin + Rain + JulianDay) *p*^B^ (Year + TempMin + Rain + JulianDay)	18	4,046.60	7.44	0.02
1 km	ψ^A^ (Year + HSF_1 km_ + HSM_1 km_) ψ^BA^ = ψ^Ba^ (Year + HSF_1 km_ + HSM_1 km_) *p*^A^ (Year + TempMin + Rain + JulianDay) *p*^B^ (Year + TempMin + Rain + JulianDay)	18	4,031.98	0.00	0.88
	ψ^A^ (Year + Decid12_1 km_ + Mixed12_1 km_ + Conif12_1 km_) ψ^BA^ = ψ^Ba^ (Year + Decid12_1 km_ + Mixed12_1 km_ + Conif12_1 km_) *p*^A^ (Year + TempMin + Rain + JulianDay) *p*^B^ (Year + TempMin + Rain + JulianDay)	20	4,036.19	4.21	0.11
	ψ^A^ (Year + HSF_1 km_ + HSM_1 km_) ψ^BA^ = ψ^Ba^ (Year + HSF_1 km_ + HSM_1 km_) *p*^A^ (Year + TempMin + Rain + LureDay) *p*^B^ (Year + TempMin + Rain + LureDay)	18	4,041.55	9.57	0.01
3 km	ψ^A^ (Year + HSF_3 km_ + HSM_3 km_) ψ^BA^ = ψ^Ba^ (Year + HSF_3 km_ + HSM_3 km_) *p*^A^ (Year + TempMin + Rain + JulianDay) *p*^B^ (Year + TempMin + Rain + JulianDay)	18	4,014.59	0.00	0.98
	ψ^A^ (Year + HSF_3 km_ + HSM_3 km_) ψ^BA^ = ψ^Ba^ (Year + HSF_3 km_ + HSM_3 km_) *p*^A^ (Year + TempMin + Rain + LureDay) *p*^B^ (Year + TempMin + Rain + LureDay)	18	4,024.15	9.56	0.01
	ψ^A^ (Year + HSF_3 km_ + HSM_3 km_) ψ^BA^ (Year + HSF_3 km_ + HSM_3 km_) ψ^Ba^ (Year + HSF_3 km_ + HSM_3 km_) *p*^A^ (Year + TempMin + Rain + JulianDay) *p*^B^ (Year + TempMin + Rain + JulianDay)	22	4,024.40	9.81	0.01
5 km	ψ^A^ (Year + HSF_5 km_ + HSM_5 km_) ψ^BA^ = ψ^Ba^ (Year + HSF_5 km_ + HSM_5 km_) *p*^A^ (Year + TempMin + Rain + JulianDay) *p*^B^ (Year + TempMin + Rain + JulianDay)	18	4,014.51	0.00	0.85
	ψ^A^ (Year + Decid12_5 km_ + Mixed12_5 km_ + Conif12_5 km_) ψ^BA^ = ψ^Ba^ (Year + Decid12_5 km_ + Mixed12_5 km_ + Conif12_5 km_) *p*^A^ (Year + TempMin + Rain + JulianDay) *p*^B^ (Year + TempMin + Rain + JulianDay)	20	4,018.35	3.84	0.13
	ψ^A^ (Year + HSF_5 km_ + HSM_5 km_) ψ^BA^ (Year + HSF_5 km_ + HSM_5 km_) ψ^Ba^ (Year + HSF_5 km_ + HSM_5 km_) *p*^A^ (Year + TempMin + Rain + JulianDay) *p*^B^ (Year + TempMin + Rain + JulianDay)	22	4,023.02	8.50	0.01

**Table 3.** —Covariate effects estimated by multimodel inference (logit scale) from two-species occupancy models on occupancy and detection probabilities of fisher and marten. Effects were estimated for all spatial scales combined and for each spatial scale separately (radii 0.5, 1, 3, and 5 km). The 95% confidence intervals around the estimates are presented between brackets. Only estimates for which the 95% CI excluded 0 are presented. Results indicate that marten occupancy is independent of fisher presence. The probability of detecting fisher is considered independent of the presence of marten. The probability of detecting marten is considered independent of the presence and detection of fisher. HSF = Hot Spots for Fishers; HSM = Hot Spots for Martens.

Parameter	Variable	All	0.5 km	1 km	3 km	5 km
Fisher occupancy	*(none)*					
Marten occupancy	HSF				−0.69 [−1.24, −0.14]	
	HSM			0.81 [0.07, 1.55]	1.44 [0.79, 2.09]	1.26 [0.14, 2.38]
Fisher detection probability	Rain	0.13 [0, 0.26]				
Marten detection probability	JulianDay	−0.22 [−0.35, −0.1]				
	Year	0.25 [0.05, 0.45]				

Comparisons across all spatial grain sizes indicated that models including HSF and HSM within 3- and 5-km radii equally shared most of the support (Table 2). These models considered site occupancy by martens to be independent of fisher presence, and included the effects of year, minimum temperature, amount of rain, and Julian date on detection probability. The model ranking third consisted of the quantities of deciduous, mixedwood, and coniferous stands > 12 m within a 5-km radius, but had low support (Akaike weight = 0.07, Table 2). HSF and HSM had as much weight at the 3-km grain size around the camera traps as at the 5-km grain sizes in explaining site occupancy by martens. Multimodel inference indicated that none of the habitat variables explained site occupancy by either species when all spatial grain sizes were considered (Table 3).

**Fig. 2.**
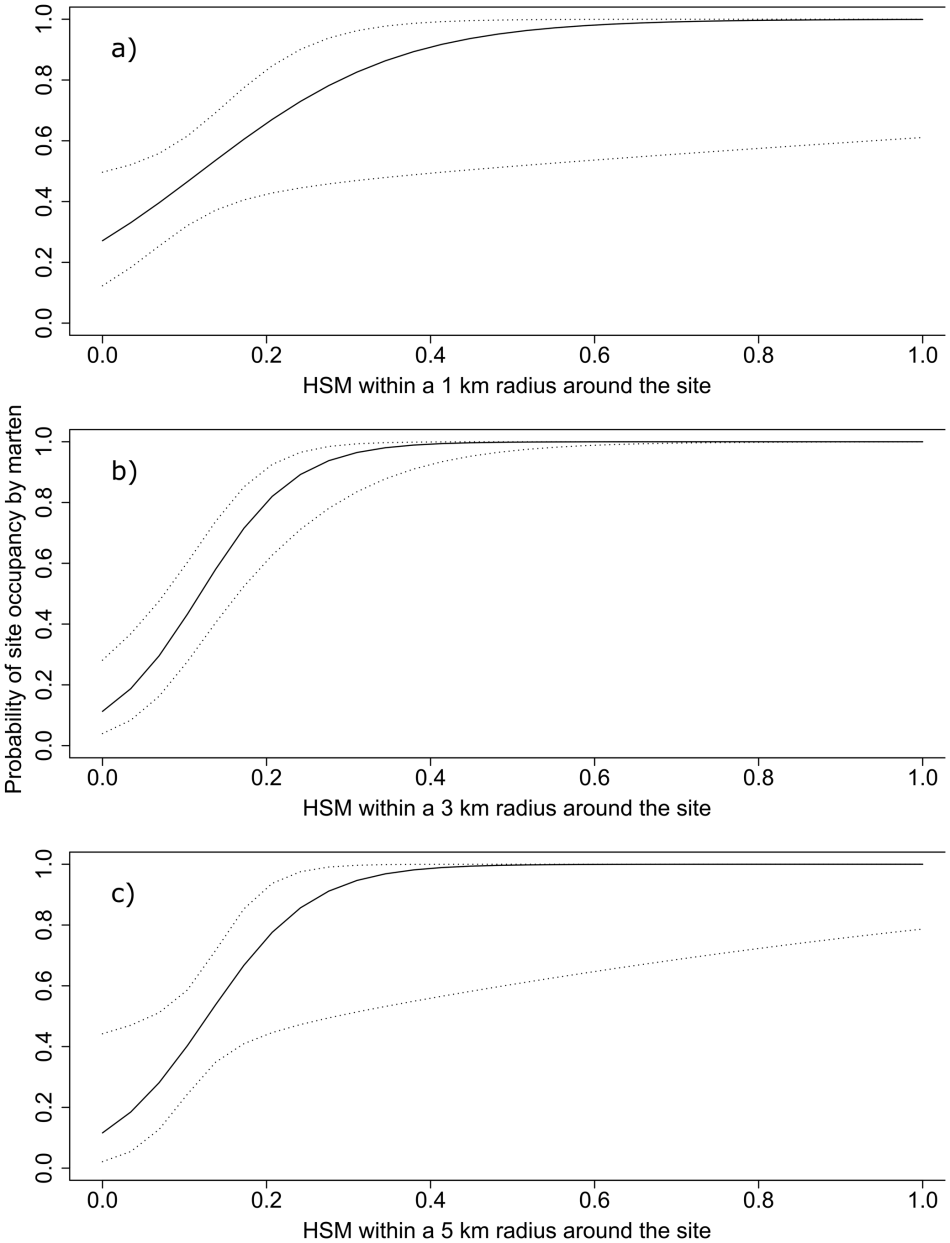
—Model-averaged probability of site occupancy by marten as a function of the quantity of habitat hotspots for marten (Hot Spots for Martens [HSM], consisting of mixedwood or coniferous stands taller than 12 m and >90 years old, with a canopy closure ≥ 60%), as defined by local knowledge, within radii of 1 km (A), 3 km (B), and 5 km (C) around the site, in the falls of 2015 and 2016 in western Québec, Canada. These results were obtained by considering each spatial scale separately.

**Fig. 3.**
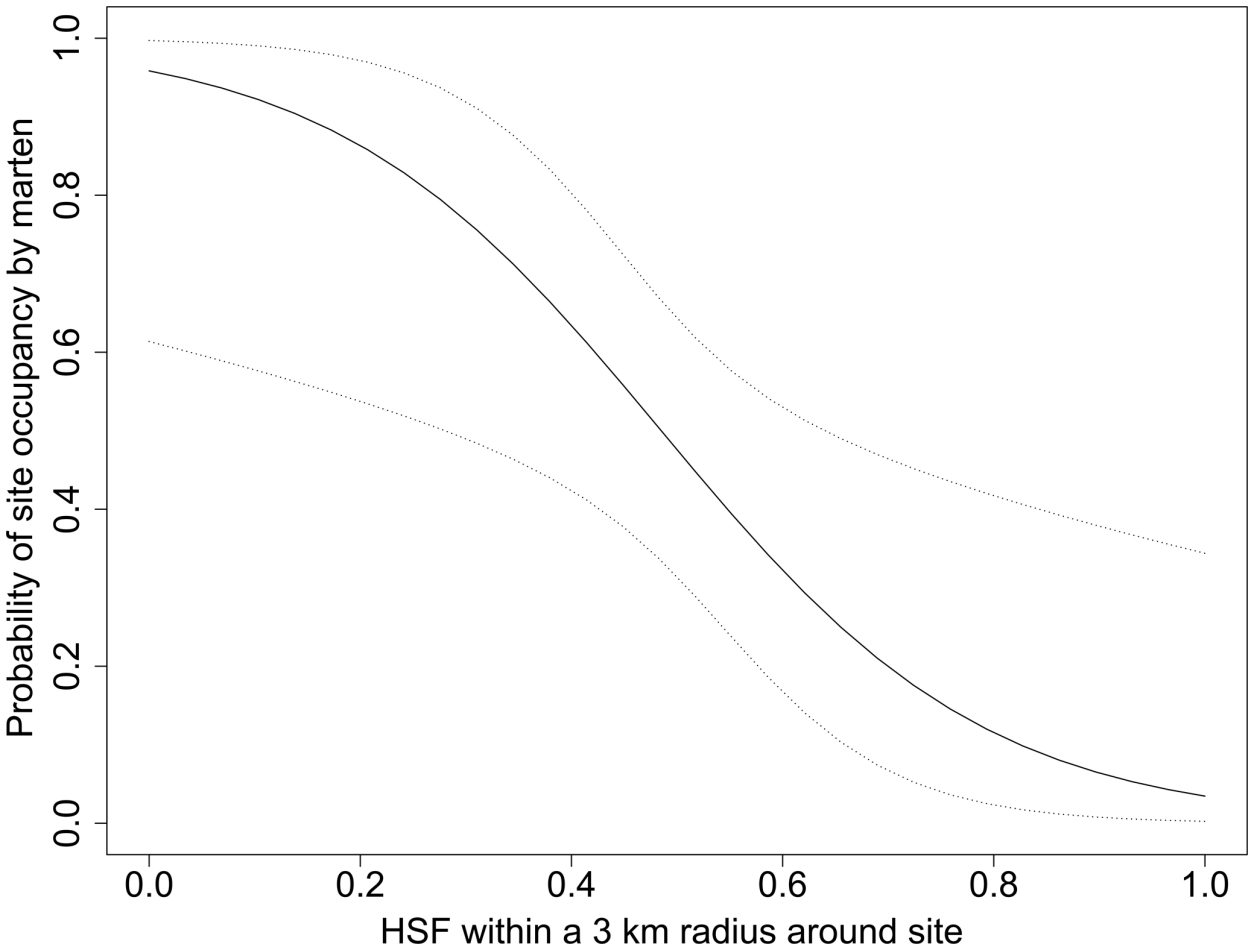
—Model-averaged probability of site occupancy by marten as a function of the quantity of habitat hotspots for fisher (Hot Spots for Fishers [HSF], consisting of coniferous or mixedwood stands taller than 7 m with a canopy closure ≥ 25%), incorporating trappers’ local knowledge, within a 3-km radius around the site in fall 2015 and 2016 in western Québec, Canada. These results were obtained by considering the 3-km spatial grain size separately.

**Fig. 4.**
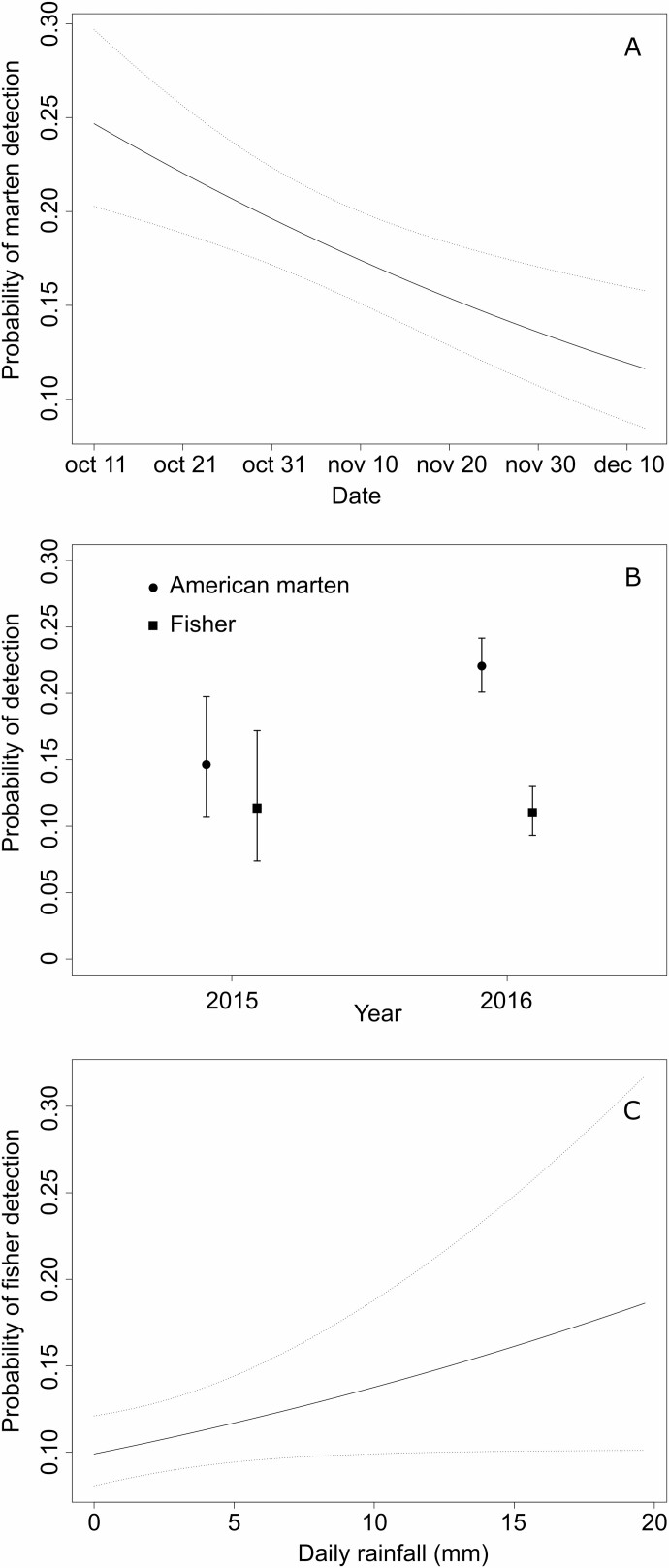
—Model-averaged probability of marten detection as a function of JulianDay (calendar date, A), marten and fisher detection as a function of year (B), and fisher detection as a function of quantity of daily rainfall (mm) (C), in falls 2015 and 2016 in western Québec, Canada. Note that all models constrained marten detection probability to be independent of fisher presence and detection, whereas fisher detection probability was independent of marten presence.

When considering the models independently for each spatial grain size, however, the probability of a site being occupied by martens (ψ^BA^ = ψ^Ba^) increased with the availability of habitat hotspots for martens (HSM) within a radius of 1, 3, and 5 km (Table 3, Fig. 2), but decreased with the availability of habitat hotspots for fishers (HSF) within a 3-km radius (Table 3, Fig. 3). Marten detection was very high with at least 20% of hotspots for martens in a radius of 3 or 5 km. In both years, the probability of detecting martens decreased throughout the sampling period in each year (Fig. 4A), but was greater in 2016 than 2015 (Fig. 4B). None of the occupancy variables considered to explain the presence of fishers had support, regardless of the spatial grain size. The probability of detecting fishers increased with the quantity of daily rainfall (Fig. 4C).

The occupancy probabilities of the species did not vary with road density, edge density, or forest habitats higher than 12 m (deciduous, mixed, or coniferous) at all spatial grain sizes investigated. The minimum temperature and the number of days since a lure was applied had no effect on the detection probabilities of the two species.

## Discussion

### Species co-occurrence

During the fall, site occupancy by American martens in temperate deciduous forests of Québec was independent of fisher site occupancy. This result suggests that fishers did not competitively exclude martens at the spatial grain size of our sampling stations. This result is contrary to Fisher et al. (2013) who found that martens and fishers select different winter habitat types in the Rocky Mountains of Central Alberta where the two species responded differently to habitat fragmentation. However, studies conducted in the states of New York, Wisconsin, and Maine did not observe spatial segregation between martens and fishers (Gompper et al. 2016; Manlick et al. 2017; Evans and Mortelliti 2022). The discrepancy between the results of studies conducted in eastern North America (Gompper et al. 2016; Manlick et al. 2017; Evans and Mortelliti 2022; this study) and in western North America (Fisher et al. 2013) may either reveal differences in space use patterns by the two species across their range or differences in the analytical approaches used. Indeed, Fisher et al. (2013) is the only study that did not account for imperfect species detection. Differences in snow conditions (Raine 1983; Pauli et al. 2022) could also explain this discrepancy but should be further explored in our study area by extending the monitoring period throughout the winter. Evans and Mortelliti (2022) did not detect a negative impact of snow depth on fisher in Maine but highlighted that improvements on model sinking depth and formation of icy crusts may provide valuable insight.

Apart from spatial exclusion, other intraguild competition mechanisms, such as temporal niche partitioning, resource partitioning, and population densities, should be evaluated to understand the interactions between the two sympatric species in eastern North America (Amarasekare 2003; Kautz et al. 2021). Sharing the same ecological niche implies sharing common food resources (Zielinski and Duncan 2004; Manlick et al. 2017), which could be a source of subsequent interference competition (Donadio and Buskirk 2006). In Wisconsin, fishers are an important cause of mortality for martens (McCann et al. 2010). In our study region, because of trapping, it is particularly difficult to distinguish between interspecific killing and opportunistic consumption of carcasses. Indeed, fishers can eat marten carcasses that have been trapped in active trapping areas. The diet of fishers should be further documented outside the trapping period to evaluate fisher predation on martens.

### Variables influencing marten site occupancy

HSM was a good predictor of marten habitat use in the fall. Marten occupancy increased with the proportion of habitat hotspots at distances of 1, 3, and 5 km from a given sampling site. HSM emphasizes the importance for martens of coniferous stands in temperate forests otherwise dominated by deciduous stands (Thomasma 1996; McCann et al. 2014). Martens responded negatively to HSF which, compared to HSM, had slightly lower stands (≥7 m vs. ≥12 m), less canopy closure (≥25% vs. ≥60%), and all ages (vs. ≥90 years). The difference in height between the two indices is small, since the 7- to 12-m stands included in HSF represent only an average of 4–5% of the habitat. The difference in age between HSF and HSM highlights the importance of old-growth stands (≥90 years) for marten in temperate deciduous forests. Marten select several habitat structures that are characteristic of older forests (e.g., rootballs and wide-diameter snags), but these characteristics can also be retained with appropriate sylvicultural treatments in some managed forests (Porter et al. 2005). Structural complexity increases with stand age due to natural successional processes such as tree senescence and creation of canopy gaps by disturbances (Powell et al. 2003; Drever et al. 2006; Després et al. 2017), hence creating conditions favorable to martens (Pauli et al. 2022).

The availability of resting sites is also important for marten habitat use (Gilbert et al. 2017). Mature forests are structurally more complex than younger stands, and thus more likely to include marten resting sites (Joyce 2013). Snow-tracking data from Ontario have shown the importance for martens of coniferous composition, tree size, coarse woody debris, and canopy closure (Bowman and Robitaille 2005). Such relationships could be established in stand inventory data from Quebec to better integrate structural attributes into the marten habitat suitability index.

Marten site occupancy in temperate deciduous forests was more strongly related to the proportion of habitat hotspots at the larger grain size (3- and 5-km radii) than at the grain size corresponding to known home range of martens in similar regions (1-km radii). Similar patterns were observed in the boreal forest (Cheveau et al. 2013). The higher importance of hotspots at larger spatial grain sizes could be explained by habitat heterogeneity increasing with the size of the spatial grain. Both the 3- and 5-km-radius grain sizes had a similar importance. The effect of HSM on marten site occupancy was greater at 3 km than at either 1 or 5 km. Thus, habitat hotspots for martens should consider not only stand quality, but also the quality of neighboring stands within a 3-km radius.

We did not find a relationship between marten site occupancy and fragmentation variables (edge density, road density) at the spatial grain sizes we investigated. This result is consistent with theoretical and empirical studies on terrestrial vertebrates that found that organisms are first affected by net habitat loss before responding to habitat fragmentation (Andrén and Andren 1994; Fahrig 1997, 2003), unless the amount of residual habitat after disturbance reaches some threshold value (often less than 30%) of its original proportion in the landscape. Interestingly, the amount of residual habitat was above this threshold in the fragmented landscapes we studied (Supplementary Data SD2).

### Variables influencing fishers site occupancy

Over two consecutive years, both fishers and martens were detected in two-thirds of our sites. However, fisher detections were more variable between years than marten detections. The number of sites where cameras detected at least one fisher was similar between years. Yet, fisher detections did not occur on the same sites between years, suggesting greater mobility by fishers than by martens. This difference could perhaps reflect winter harvesting by trapping or colonization of new sites by young fishers that dispersed during the summer 2016. None of the habitat variables that we considered explained site occupancy by fishers. In our study area, habitat availability at the landscape scale does not seem to be limiting for fishers in the temperate deciduous forest during the fall. Tully (2006) found that radio-collared fishers selected habitat corresponding to stands with larger trees and a higher diversity of tree species. Immature and young stands with small trees were also used, but to a lesser extent (Tully 2006). Sauder and Rachlow (2015) reported that fishers prefer heterogeneous habitats within the main area of use of their home range. Whereas telemetry studies often extend over several months or seasons, our study was limited to the fall (Tully 2006; Kordosky et al. 2021). A better assessment of fisher habitat use in our study area would require data collected during winter and spring, when movements are limited by snow and young rearing (Gilbert et al. 2017).

### Variables influencing detection probabilities

The detection probabilities of the two species did not vary with the number of days following the application of the olfactory lures. The odor of the lure, combined with that of the carcass and the skin, did not appear to fade, even during the longest period between two lure applications (26 days). However, the decreasing probability of detecting martens with the progression of the season (Julian date) may be a consequence of trapping activity. Unpublished data from the Quebec Ministry of Forests, Wildlife and Parks indicate that the largest proportion of marten and fisher captures occurred during November, which corresponds to the middle of our camera monitoring period. Fisher detection probabilities did not vary across the season, although this result is difficult to interpret.

The detection probability for martens was higher in 2016 than in 2015, which may reflect factors not measured in our study, such as abundance of prey, predators, and competitors, or snow accumulation on the ground, which can alter their foraging behavior (Jensen et al. 2012; Sweitzer and Furnas 2016). Our weekly visits to the sites revealed that the onset of winter was earlier in 2016 than in 2015. Snow accumulation on the ground was particularly high (up to about 60 cm) as early as mid-November 2016, while the ground surface was still visible in mid-December 2015. Alternatively, individuals could have become used to the presence of carcasses at the same sites in both years, which could have generated a “preferred” food source, when the search for live prey became more energy-consuming in the snow. Given their smaller home range size, martens may more likely display greater fidelity toward an alternative food source at our sampling stations than do fishers that have a much larger home range. Contrary to our hypothesis, the probability of detecting fishers in the fall increased with rainfall. The meteorological factors that affect mesocarnivore activity are still poorly known. According to the thermal cost hypothesis, activity should be correlated with environmental conditions favorable to energy conservation (Zielinski 2000). We expected that rainfall in the fall would reduce the movement of individuals of both species, which would seek to limit the risk of hypothermia. Further, we hypothesized that rain would limit the dispersion of lure and bait odors, which depend upon concentration, wind speed, atmospheric stability, and vegetation (Pawlina and Proulx 1999).

Nevertheless, akin to our results, Tully (2006) reported higher daily catch rates of fishers with higher precipitation and lower temperatures. Although we did not find any relationship between minimum air temperature and detection probability of fishers, the positive effect of rainfall could reflect preferential use of baits when energy costs were higher in cool rainy days than during dry weather conditions. There is a need to document daily movements of fishers in relation to rainfall, snow accumulation on the ground, and snow cover load-bearing capacity.

### Conclusions

Our study suggests that in temperate deciduous forests of Québec, where the two species are sympatric, site occupancy by martens is not affected by fisher presence. Availability of old and dense coniferous stands increased the habitat use of martens while habitat hotspots for martens developed from the empirical knowledge of trappers is associated to marten occupancy patterns. The characteristics of habitat hotspots refine the habitat suitability model for American martens in the temperate deciduous forest and highlight the importance of combining several attributes of stand structure to describe marten habitat, including stand composition, age, and canopy closure. In contrast, site occupancy by fishers in the fall did not vary with any habitat characteristics. This result is thus consistent with the empirical knowledge of trappers that fishers are generalists in this region.

## Supplementary Data

Supplementary data are available at *Journal of Mammalogy* online.

Supplementary Data SD1.—Two-species site occupancy models evaluating effects of covariates on the probability of site occupancy by fisher (ψ^A^), and by marten, when fisher is present (ψ^BA^) or absent (ψ^Ba^) in temperate forests of western Quebec. Note that each series of models in the table was run with either marten occupancy independent of fisher presence (ψ^BA^ = ψ^Ba^, 25 models) or dependent on fisher presence (ψ^BA^ ≠ ψ^Ba^, 25 models), for a total of 50 candidate models that included variables not strongly correlated with one another (|*r*| < 0.7).

Supplementary Data SD2.—Variation among sites in the proportions of water bodies (Water), as well as proportions of forest habitat by tree height class (4–6 m; 7–12 m; >12 m) and by stand dominance group (D: deciduous, M: mixedwood, C: coniferous). Values are proportions of each habitat type at each spatial grain size around the sites (radii: 0.5, 1, 3, and 5 km). Boxes represent the 1st and 3rd quartiles (interquartile distance), while the horizontal line within the box is the median. Lower and upper whiskers represent scores outside the middle 50%. Open circles are outliers beyond 1.5 times the interquartile distance.

Supplementary Data SD3.—Proportions of habitat hotspots for fisher (HSF: white boxes) and marten (HSM: gray boxes) at sampled sites, as defined by local trapper knowledge. The values represent the proportions of each habitat type at each spatial grain size around the sites (radii: 0.5, 1, 3, and 5 km) in 2015 (similar in 2016). Boxes represent interquartile distances, while the horizontal line within the box is the median. Lower and upper whiskers represent scores outside the middle 50%. Open circles are outliers beyond 1.5 times the interquartile distance.

Supplementary Data SD4.—Edge density (km/km^2^) between stands < 4 m in height and stands ≥ 4 m in height at each spatial grain size around the sites (radii: 0.5, 1, 3, and 5 km) in 2015 (similar in 2016). Boxes represent the 1st and 3rd quartiles (interquartile distance), while the horizontal line within the box is the median. Lower and upper whiskers represent scores outside the middle 50%. Open circles are outliers beyond 1.5 times the interquartile distance.

Supplementary Data SD5.—Road density index corresponding to the length of roads weighted according to their use class (km/km^2^) at each spatial grain size (radii: 0.5, 1, 3, and km) in 2015 (similar in 2016). Boxes represent the 1st and 3rd quartiles (interquartile distance), while the horizontal line within the box is the median. Lower and upper whiskers represent scores outside the middle 50%. Open circles are outliers beyond 1.5 times the interquartile distance.

Supplementary Data SD6.—Distribution of the number of sites with a given number of days with detection of fisher (a) and marten (b) in a network of 49 camera traps that were baited and monitored in western Québec, Canada, during the fall in 2015 and 2016.

gyac092_suppl_Supplementary_Data
