# Supplementary material for: Site occupancy by American martens and fishers in temperate deciduous forests of Québec
Source: J Mammal. 2022 Dec 9;104(1):159–70. doi: 10.1093/jmammal/gyac092 (PMC9936503; doi:10.1093/jmammal/gyac092)
Supplement: gyac092_suppl_Supplementary_Data [file gyac092_suppl_supplementary_data.docx]

**SUPPLEMENTARY DATA**

Supplementary Data SD1. —. Two-species site occupancy models evaluating effects of covariates on the probability of site occupancy by fisher (ψ^A^), and by marten, when fisher is present (ψ^BA^) or absent (ψ^Ba^) in temperate forests of western Quebec. Note that each series of models in the table was run with either marten occupancy independent of fisher presence (ψ^BA^ = ψ^Ba^, 25 models) or dependent on fisher presence (ψ^BA^ ≠ ψ^Ba^, 25 models), for a total of 50 candidate models that included variables not strongly correlated with one another (|r| < 0.7).

| Models | Spatial scale | Occupancy *(ψ^A^, ψ^BA^, ψ^Ba^)* | Detection *(p^A^*=*r^A^*, *p^B^*=*r^Ba^*=*r^BA^*) |
| --- | --- | --- | --- |
| Null |  | Year | Year |
| Habitat | 0.5 km | Year + Decid12_05km_ + Mixed12_05km_ + Conif12_05km_ | Year + TempMin + Rain + LureDay |
|  |  | Year + Decid12_05km_ + Mixed12_05km_ + Conif12_05km_ | Year + TempMin + Rain + JulianDay |
|  | 1 km | Year + Decid12_1km_ + Mixed12_1km_ + Conif12_1km_ | Year + TempMin + Rain + LureDay |
|  |  | Year + Decid12_1km_ + Mixed12_1km_ + Conif12_1km_ | Year + TempMin + Rain + JulianDay |
|  | 3 km | Year + Decid12_3km_ + Mixed12_3km_ + Conif12_3km_ | Year + TempMin + Rain + LureDay |
|  |  | Year + Decid12_3km_ + Mixed12_3km_ + Conif12_3km_ | Year + TempMin + Rain + JulianDay |
|  | 5 km | Year + Decid12_5km_ + Mixed12_5km_ + Conif12_5km_ | Year + TempMin + Rain + LureDay |
|  |  | Year + Decid12_5km_ + Mixed12_5km_ + Conif12_5km_ | Year + TempMin + Rain + JulianDay |
| Fragmentation | 0.5 km | Year + Roads_05km_ + Edges_05km_ | Year + TempMin + Rain + LureDay |
|  |  | Year + Roads_05km_ + Edges_05km_ | Year + TempMin + Rain + JulianDay |
|  | 1 km | Year + Roads_1km_ + Edges_1km_ | Year + TempMin + Rain + LureDay |
|  |  | Year + Roads_1km_ + Edges_1km_ | Year + TempMin + Rain + JulianDay |
|  | 3 km | Year + Roads_3km_ + Edges_3km_ | Year + TempMin + Rain + LureDay |
|  |  | Year + Roads_3km_ + Edges_3km_ | Year + TempMin + Rain + JulianDay |
|  | 5 km | Year + Roads_5km_ + Edges_5km_ | Year + TempMin + Rain + LureDay |
|  |  | Year + Roads_5km_ + Edges_5km_ | Year + TempMin + Rain + JulianDay |
| Local knowledge | 0.5 km | Year + HSF_05km_ + HSM_05km_ | Year + TempMin + Rain + LureDay |
|  |  | Year + HSF_05km_ + HSM_05km_ | Year + TempMin + Rain + JulianDay |
|  | 1 km | Year + HSF_1km_ + HSM_1km_ | Year + TempMin + Rain + LureDay |
|  |  | Year + HSF_1km_ + HSM_1km_ | Year + TempMin + Rain + JulianDay |
|  | 3 km | Year + HSF_3km_ + HSM_3km_ | Year + TempMin + Rain + LureDay |
|  |  | Year + HSF_3km_ + HSM_3km_ | Year + TempMin + Rain + JulianDay |
|  | 5 km | Year + HSF_5km_ + HSM_5km_ | Year + TempMin + Rain + LureDay |
|  |  | Year + HSF_5km_ + HSM_5km_ | Year + TempMin + Rain + JulianDay |


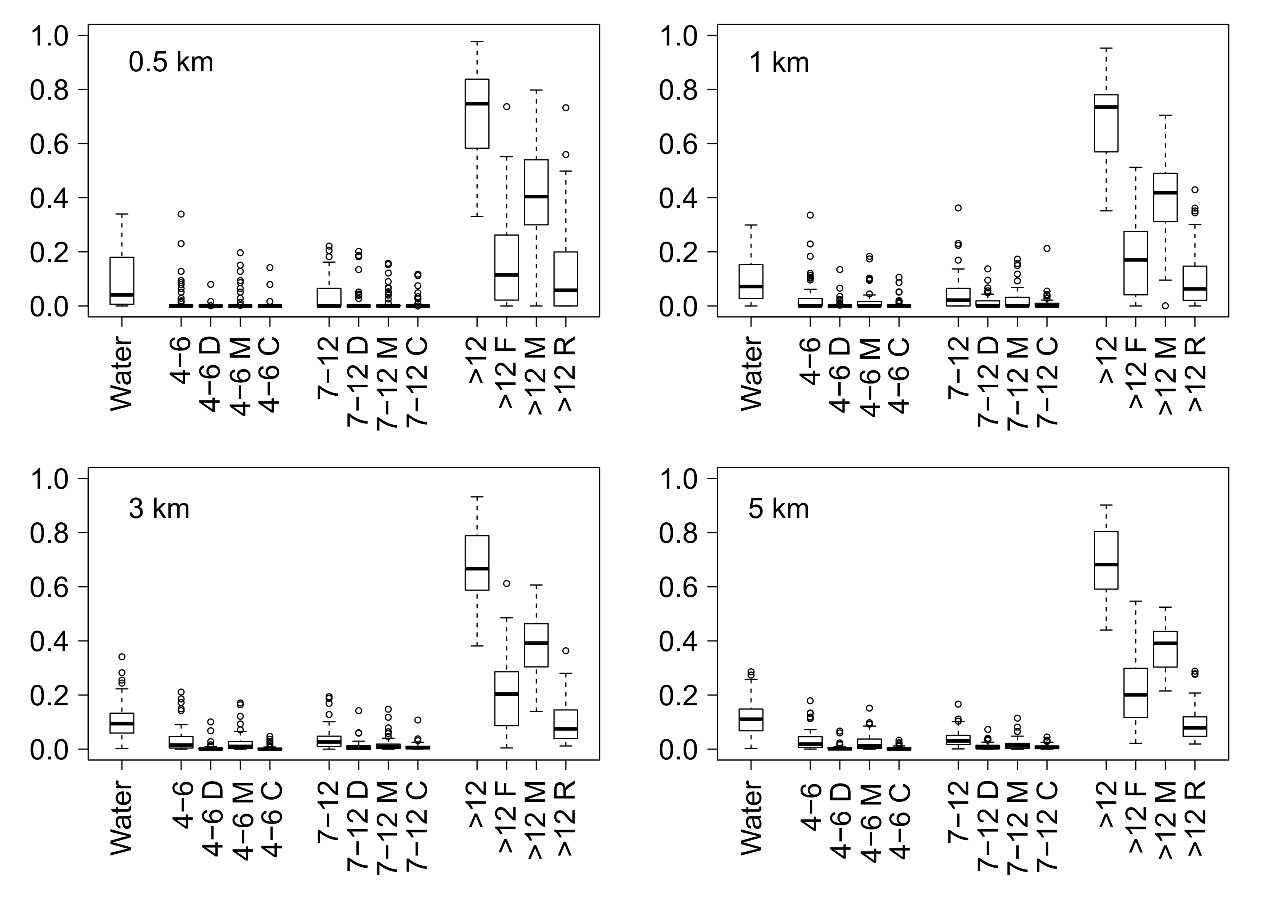


Supplementary Data SD2. — Variation among sites in the proportions of water bodies (Water), as well as proportions of forest habitat by tree height class (4-6 m; 7-12 m; > 12 m) and by stand dominance group (D: deciduous, M: mixedwood, C: coniferous). Values are proportions of each habitat type at each spatial grain size around the sites (radii: 0.5, 1, 3 and 5 km). Boxes represent the 1st and 3rd quartiles (interquartile distance), while the horizontal line within the box is the median. Lower and upper whiskers represent scores outside the middle 50%. Open circles are outliers beyond 1.5 times the interquartile distance.


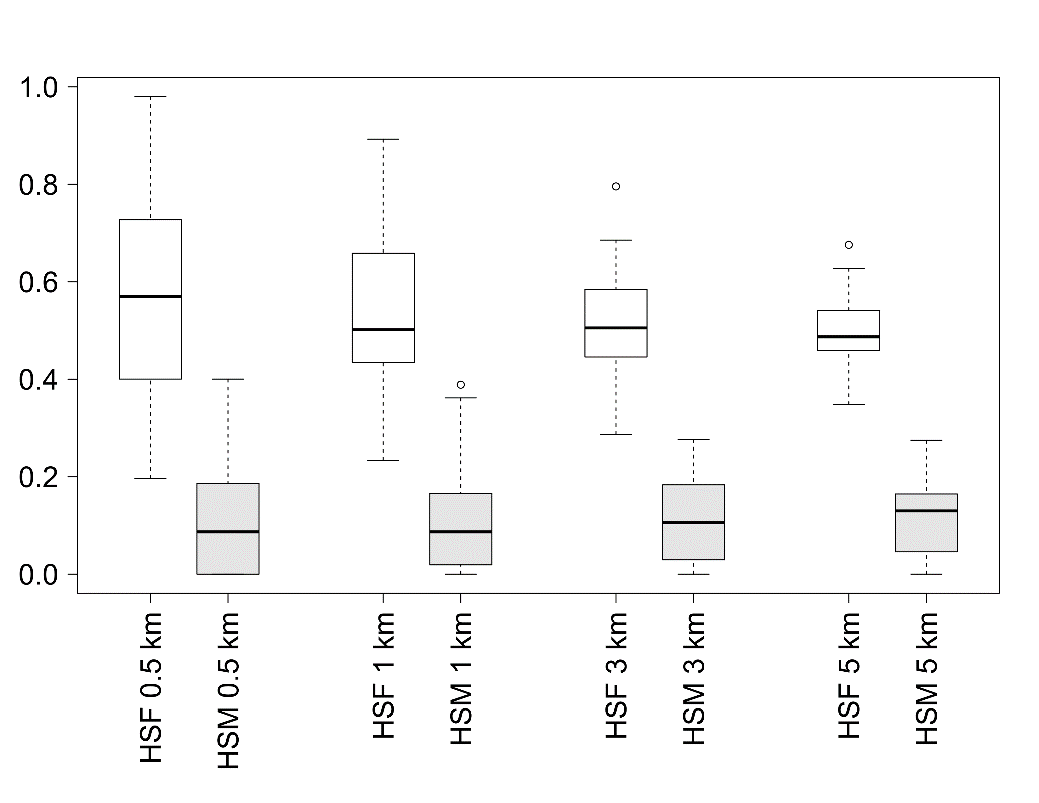


Supplementary Data SD3. — Proportions of habitat hotspots for fisher (HSF: white boxes) and marten (HSM: gray boxes) at sampled sites, as defined by local trapper knowledge. The values represent the proportions of each habitat type at each spatial grain size around the sites (radii: 0.5, 1, 3 and 5 km) in 2015 (similar in 2016). Boxes represent interquartile distances, while the horizontal line within the box is the median. Lower and upper whiskers represent scores outside the middle 50%. Open circles are outliers beyond 1.5 times the interquartile distance.


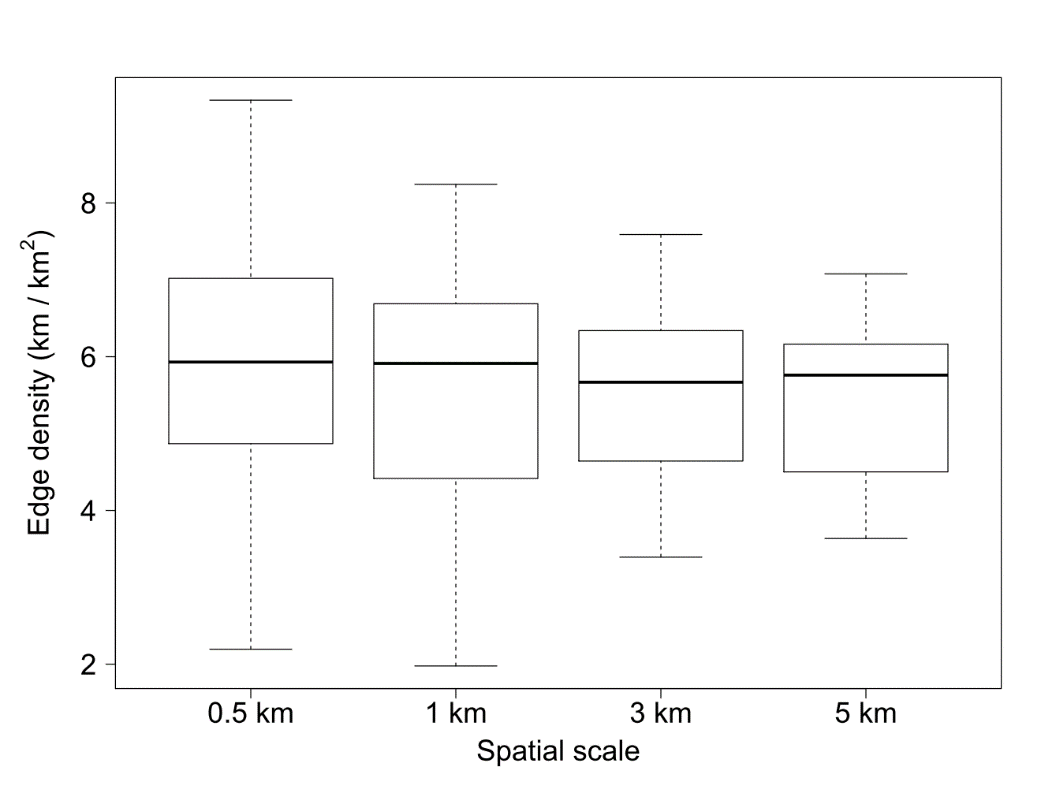


Supplementary Data SD4. — Edge density (km/km2) between stands < 4 m in height and stands ≥ 4 m in height at each spatial grain size around the sites (radii: 0.5, 1, 3 and 5 km) in 2015 (similar in 2016). Boxes represent the 1st and 3rd quartiles (interquartile distance), while the horizontal line within the box is the median. Lower and upper whiskers represent scores outside the middle 50%. Open circles are outliers beyond 1.5 times the interquartile distance.


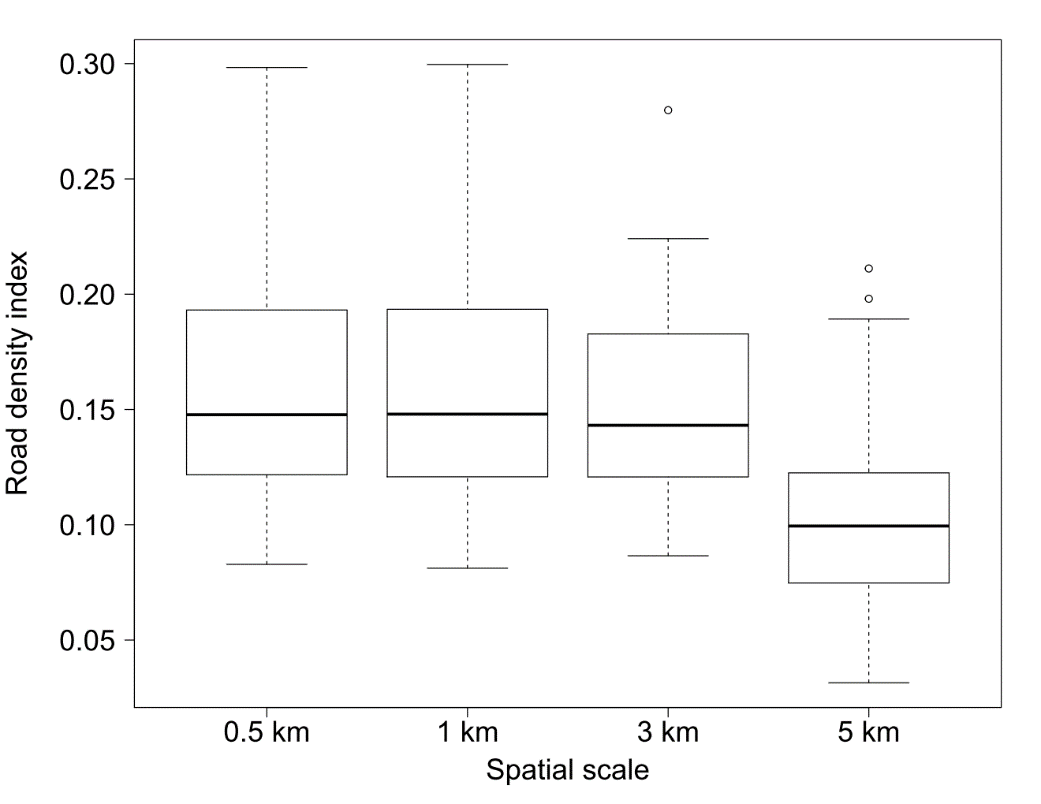


Supplementary Data SD5. — Road density index corresponding to the length of roads weighted according to their use class (km/km^2^) at each spatial grain size (radii : 0.5, 1, 3 and km) in 2015 (similar in 2016). Boxes represent the 1st and 3rd quartiles (interquartile distance), while the horizontal line within the box is the median. Lower and upper whiskers represent scores outside the middle 50%. Open circles are outliers beyond 1.5 times the interquartile distance.


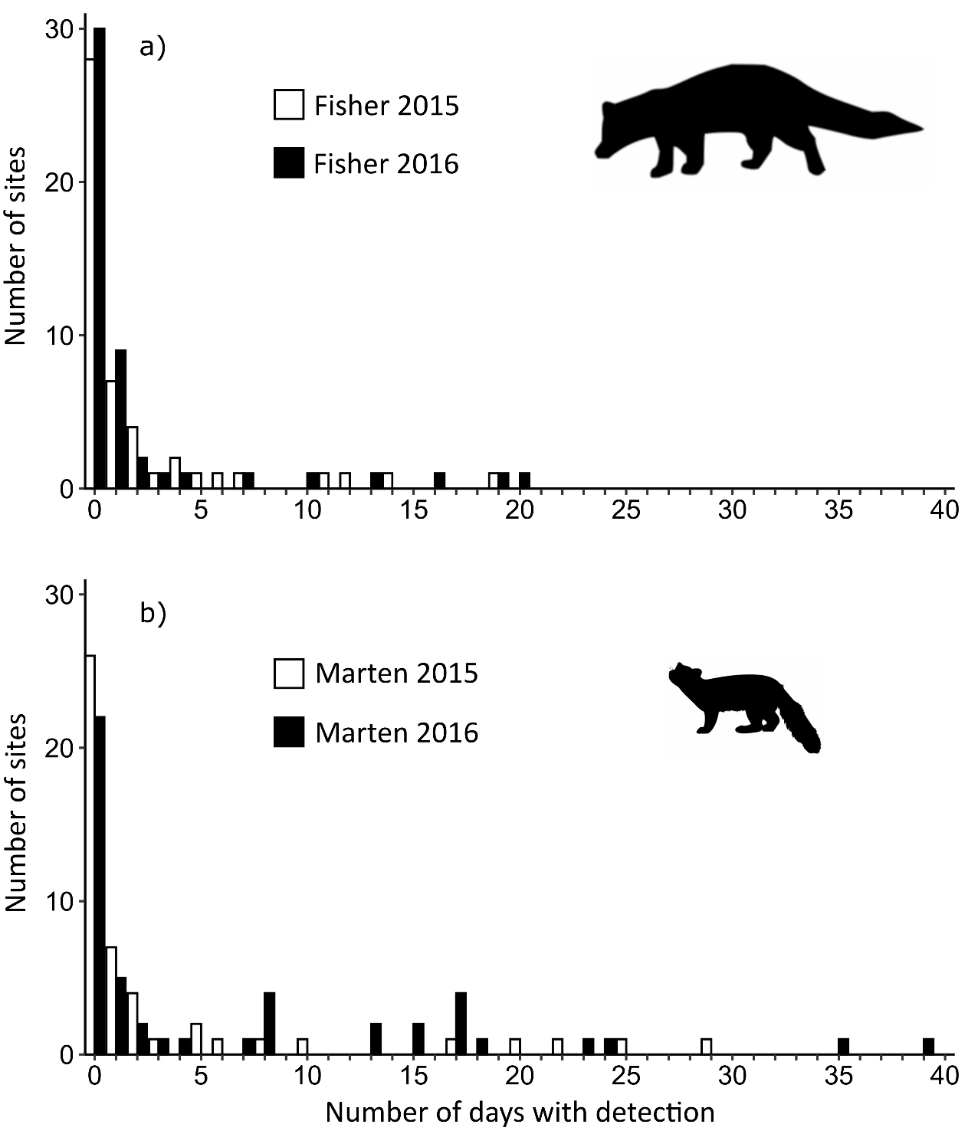


Supplementary Data SD6. — Distribution of the number of sites with a given number of days with detection of fisher (a) and marten (b) in a network of 49 camera traps that were baited and monitored in western Québec, Canada during the fall in 2015 and 2016.
